# Documenting the short‐tailed albatross (*Phoebastria albatrus*) clades historically present in British Columbia, Canada, through ancient DNA analysis of archaeological specimens

**DOI:** 10.1002/ece3.9116

**Published:** 2022-07-31

**Authors:** Thomas C. A. Royle, Eric. J. Guiry, Hua Zhang, Lauren T. Clark, Shalegh M. Missal, Sophie A. Rabinow, Margaretta James, Dongya Y. Yang

**Affiliations:** ^1^ Ancient DNA Laboratory, Department of Archaeology Simon Fraser University Burnaby British Columbia Canada; ^2^ School of Archaeology and Ancient History University of Leicester Leicester UK; ^3^ Department of Anthropology Trent University Peterborough Ontario Canada; ^4^ Department of Anthropology University of British Columbia Vancouver British Columbia Canada; ^5^ Department of Archaeology University of Cambridge Cambridge UK; ^6^ Land of Maquinna Cultural Society Mowachaht/Muchalaht First Nation Tsaxana (Gold River) British Columbia Canada

**Keywords:** ancient DNA, archaeo‐ornithology, genetic structure, historical biogeography, historical ecology, short‐tailed albatross, zooarchaeology

## Abstract

The short‐tailed albatross (*Phoebastria albatrus*) is a threatened seabird whose present‐day range encompasses much of the North Pacific. Within this species, there are two genetic clades (Clades 1 and 2) that have distinctive morphologies and foraging ecologies. Due to a global population collapse in the late 19th and early 20th centuries, the frequency of these clades among the short‐tailed albatross population that historically foraged off British Columbia, Canada, is unclear. To document the species' historical genetic structure in British Columbia, we applied ancient DNA (aDNA) analysis to 51 archaeological short‐tailed albatross specimens from the Yuquot site (Borden site number: DjSp‐1) that span the past four millennia. We obtained a 141 bp cytochrome *b* sequence from 43 of the 51 (84.3%) analyzed specimens. Analyses of these sequences indicate 40 of the specimens belong to Clade 1, while 2 belong to Clade 2. We also identified a single specimen with a novel cytochrome *b* haplotype. Our results indicate that during the past four millennia most of the short‐tailed albatrosses foraging near Yuquot belonged to Clade 1, while individuals from other lineages made more limited use of the area. Comparisons with the results of previous aDNA analyses of archaeological albatrosses from Japanese sites suggest the distribution of Clades 1 and 2 differed. While both albatross clades foraged extensively in the Northwest Pacific, Clade 1 albatrosses appear to have foraged along the west coast of Vancouver Island to a greater extent. Due to their differing distributions, these clades may be exposed to different threats.

## Graphical abstract

We applied ancient DNA analysis to 51 short‐tailed albatross specimens from the Yuquot archaeological site, British Columbia. Our results indicate that during the past four millennia most of the short‐tailed albatrosses foraging near Yuquot belonged to Clade 1.

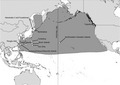

## 1 INTRODUCTION

**FIGURE 1:**
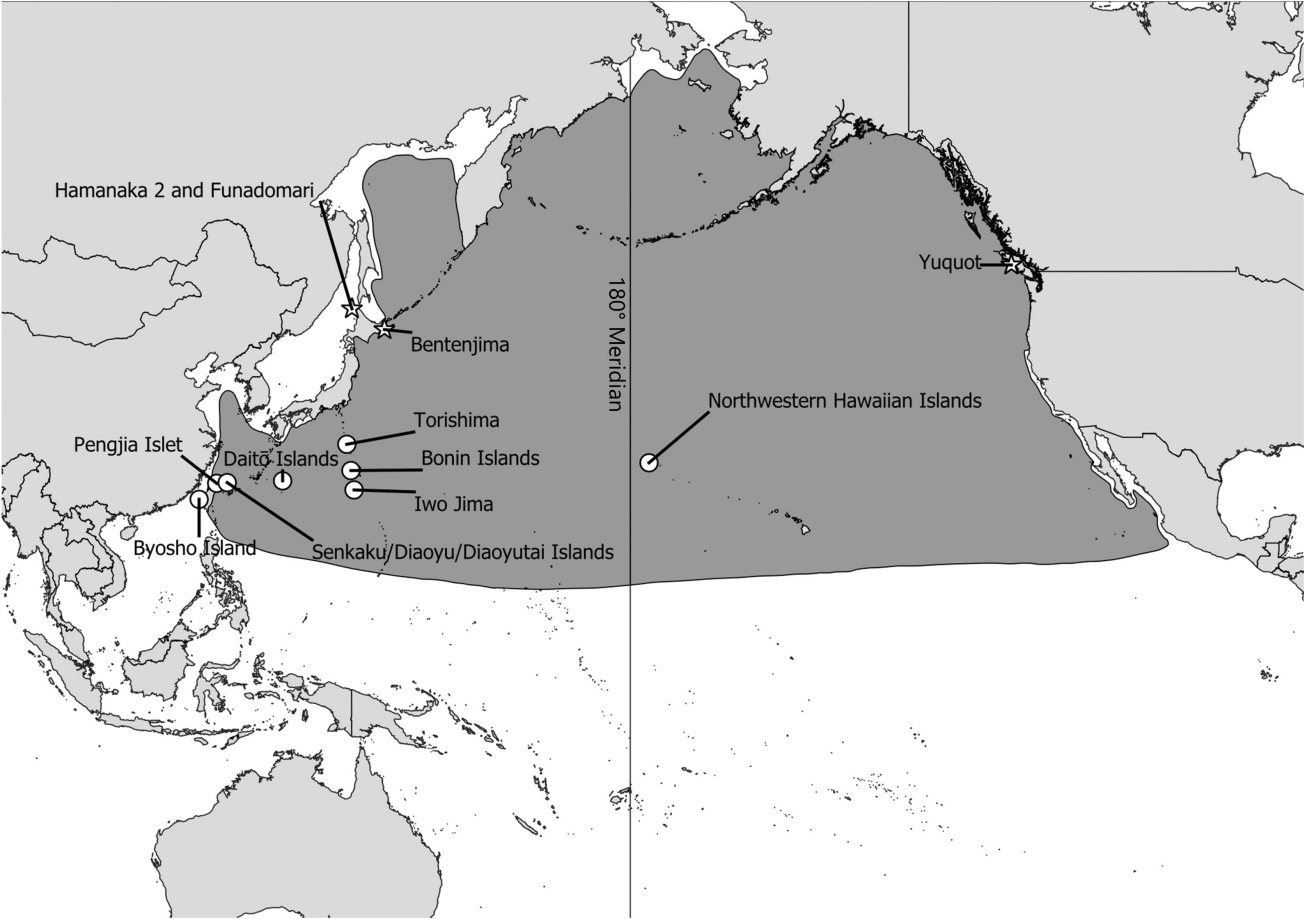
Distribution of the short‐tailed albatross (shaded area). Islands where the species currently breeds or is known to have historically bred are denoted with circles. Yuquot and other archaeological sites mentioned in the text are labeled with stars. Range data are from BirdLife International (2018).

The short‐tailed (or Stellar's) albatross (*Phoebastria albatrus*) is a large colonial breeding seabird native to the North Pacific (Austin Jr., 1949; Hasegawa & DeGange, 1982; McDermond & Morgan, 1993) (Figure 1). Today, short‐tailed albatrosses breed primarily on Torishima in the Izu Islands and Minamikojima and Kitakojima in the Senkaku/Diaoyu/Diaoyutai Islands, with limited breeding or attempted breeding also occurring on Mukojima and Nakodojima in the Bonin Islands as well as Midway and Kure Atolls in the Northwestern Hawaiian Islands (Figure 1) (Deguchi et al., 2012, 2017; Hasegawa & DeGange, 1982; U.S. Fish and Wildlife Service, 2020). In addition to these islands, breeding colonies are also known to have been historically present on a number of other islands in the Northwest Pacific (Figure 1) (Austin Jr., 1949; Hasegawa & DeGange, 1982; U.S. Fish and Wildlife Service, 2008). Although primarily breeding on islands located to the west of the 180° meridian, the foraging range of the short‐tailed albatross encompasses much of the North Pacific, including the coastal waters of British Columbia, Canada (Figure 1) (Austin Jr., 1949; Hasegawa & DeGange, 1982; McDermond & Morgan, 1993).

Historical records indicate that prior to the 20th century short‐tailed albatross was one of the most abundant seabirds in British Columbia (Carter & Sealy, 2014). For example, in 1891, Fannin (1891, p. 6) noted the species was “tolerably common” along the west and east coasts of Vancouver Island and was also present in the Strait of Juan de Fuca. Similarly, in 1904, Kermode (1904, p. 10) observed that he “found this species quite common in the Pacific Ocean near Cape Beal.” The historical presence and abundance of short‐tailed albatross in British Columbia is also reflected in the province's archaeological record. Skeletal remains from the species have been recovered from post‐ and pre‐European contexts at numerous Indigenous archaeological sites in coastal British Columbia (Crockford, 2003). At some archaeological sites, such as the Maplebank (Borden site number: DcRu‐12) (Crockford et al., 1997) and Yuquot (Borden site number: DjSp‐1) (McAllister, 1980) sites, short‐tailed albatrosses represent a large fraction of the identified bird remains. This suggests the species was not only historically abundant in British Columbian waters but was also an important source of food and/or raw materials for some Indigenous peoples in the province. Abundant short‐tailed albatross remains have also been recovered from archaeological sites on the Aleutian and Channel Islands in Alaska and California, further attesting to the species' former abundance along North America's Pacific Coast (Causey et al., 2005; Porcasi, 1999a).

During the late 19th and early 20th centuries, breeding short‐tailed albatrosses were the focus of an industrial‐scale hunt that resulted in a global population collapse (Austin Jr., 1949; Hasegawa & DeGange, 1982). Hunting‐driven population losses were exacerbated by volcanic eruptions in 1902 and 1939 that disturbed the species' nesting grounds on Torishima (Austin Jr., 1949; Hasegawa & DeGange, 1982). Predation of short‐tailed albatrosses and their eggs by introduced cats and rats may have also contributed to the species' decline (Hasegawa, 1979). As a result of this population collapse, the species has rarely been sighted in British Columbia since the early 20th century and is now classified by the Canadian government as threatened (COSEWIC, 2013). Between 1907 and 1958, there were no short‐tailed albatross sightings in the province (Carter & Sealy, 2014). Since 1958, the species has only been sporadically observed in the province, with only 85 sightings occurring in or near British Columbian waters since 1960 (COSEWIC, 2013).

On account of the species' global population collapse, nothing is known about the genetic structure of the short‐tailed albatross population that historically foraged off the coast of British Columbia. Genetic studies of modern and archaeological short‐tailed albatrosses from Japan have identified two genetic clades (Clades 1 and 2) that tend to breed on different islands (Eda et al., 2006; Eda et al., 2012; Kuro‐o et al., 2010). Currently, albatrosses belonging to Clade 1 are only known to breed on Torishima, while individuals from Clade 2 breed primarily on the Senkaku/Diaoyu/Diaoyutai Islands (Eda et al., 2012; Kuro‐o et al., 2010). However, due to recent immigration from the Senkaku/Diaoyu/Diaoyutai Islands, a few Clade 2 individuals also now breed on Torishima (Eda et al., 2011, 2012; Eda, Izumi, et al., 2016; Kuro‐o et al., 2010). In addition to being genetically distinct, stable isotope analysis of archaeological specimens from the Hamanaka 2 site on Rebun Island, Japan, (Figure 1) suggests Clade 1 and 2 birds have differing foraging ecologies (Eda et al., 2012). Analyses of archaeological and modern short‐tailed albatrosses also suggest these clades vary in their morphology, with both their size and beak shape differing (Eda et al., 2012, 2020). The distinctiveness of these breeding populations is maintained by within‐clade assortative mating, which limits gene flow (Eda, Izumi, et al., 2016). This assortative mating is believed to be due to prezygotic barriers, including inter‐clade differences in courtship displays and breeding season timing (Eda, Izumi, et al., 2016). Recently, (Eda et al., 2020; Yamasaki et al., 2022) have argued that the morphological, genetic, and ecological differences between these clades as well as the propensity for within‐clade assortative mating suggest they may in fact represent two cryptic species. Understanding the distribution of these clades has been identified as being critical to developing effective conservation and recovery plans for the species (Eda et al., 2020).

In recent years, ancient DNA (aDNA) analysis of archaeological, palaeontological, and taxidermied specimens has been increasingly used to document the historical genetic structure of bird species (Grealy et al., 2017). Previous studies have used aDNA analysis to document the historical genetic structure of short‐tailed albatrosses in Japan (Eda et al., 2006, 2012; Eda, Koike, & Higuchi, 2016), and a variety of other bird species including kākāpō (*Strigops habroptilus*) (Seersholm et al., 2018), passenger pigeon (*Ectopistes migratorius*) (Guiry et al., 2020; Murray et al., 2017), and eastern Moa (*Emeus crassus*) (Verry et al., 2022). In light of the success of these previous studies, we used aDNA analysis to document the historical genetic structure of short‐tailed albatrosses in British Columbia. Here, we applied aDNA analysis to a sample of 51 short‐tailed albatross specimens from the Yuquot (Figure 1) archaeological site in order to determine the clade membership of the birds represented at the site. As the analyzed specimens span the past four millennia (Dewhirst, 1980; McAllister, 1980), we also sought to determine whether any temporal shifts in the relative abundance of short‐tailed albatross clades occurred as a result of anthropogenic or natural processes. Our data indicate that for the past four millennia most of the albatrosses that foraged in the vicinity of Yuquot belonged to Clade 1 and that birds from other genetic lineages made limited use of the area.

## 2 METHODS

### 2.1 Specimens

We performed aDNA analysis on 51 skeletal remains from the Yuquot archaeological site that were identified as short‐tailed albatross through comparative morphological analyses by McAllister (1980). Yuquot is located on the southeastern tip of Nootka Island at the entrance of Nootka Sound on the west coast of Vancouver Island (Dewhirst, 1980). This locale is the ancestral home of the Mowachaht—a Nuu‐chah‐nulth people—and encompasses both a village site and broader cultural landscape. All of the remains analyzed in this study are from the village site. Excavations of the village site were undertaken in 1966 by Parks Canada's Yuquot Archaeology Project (Dewhirst, 1980). The results of these excavations indicate that the village was first inhabited by 2300 BCE and has been continuously occupied since its initial occupation (Dewhirst, 1980). The archaeological sequence at Yuquot has been divided into four stratigraphic zones: Zone I (pre‐2300–1000 BCE), Zone II (1000 BCE–800 CE), Zone III (800–1790 CE), and Zone IV (1790–1966 CE) (Dewhirst, 1980). Dates for these stratigraphic zones were established through a combination of radiocarbon dating and temporally diagnostic artifacts (Dewhirst, 1980). Our sample included specimens from each of the four stratigraphic zones represented at the site (Table S1). Table S1 provides provenience and skeletal element information for each of the analyzed specimens. Although the site's archaeological sequence terminates at 1966 CE, Yuquot continues to be occupied by members of the Mowachaht/Muchalaht First Nation, and today forms part of Yuquot Indian Reserve 1. The Yuquot faunal assemblage is currently curated by the Canadian Museum of History (Gatineau, Quebec, Canada). Permission to conduct genetic analyses on the specimens included in this study was provided by the Mowachaht/Muchalaht First Nation and Parks Canada.

### 2.2 Decontamination and DNA extraction

All pre‐PCR procedures (Decontamination, DNA extraction, and PCR setup) were conducted in a dedicated aDNA laboratory within the Department of Archaeology, Simon Fraser University (Burnaby, British Columbia, Canada), that is positively pressured and equipped with a UV HEPA air filter. Strict contamination control protocols, including the use of protective clothing, gloves, and regular cleaning of work surfaces with bleach, were followed throughout the analysis (Yang & Watt, 2005). No analyses of modern specimens or PCR amplifications have ever been performed within this laboratory.

Small 30–321 mg chunks of bone were removed from each of the archaeological specimens and decontaminated using the protocol described by Speller et al. (2012) (Table S1). In brief, to decontaminate the specimens they were immersed in a 100% commercial bleach solution (≈5% w/v NaOCl; Clorox Company, Brampton, Ontario, Canada) for 6–8 min; rinsed in distilled water for 30 sec–2 min; rinsed a second time in distilled water for 6–10 min; and UV irradiated in a crosslinker for 30–60 mins (Speller et al., 2012). The decontaminated specimens were incubated overnight at 50°C with 3–4.5 ml of lysis buffer (0.5 M EDTA [pH 8.0], 0.25% SDS, and 0.5 mg/ml proteinase K) in a rotating hybridization oven (Yang et al., 1998, 2008). DNA was extracted and purified from the lysed specimens using a modified silica‐spin column method (Yang et al., 1998, 2008). To monitor for contamination, blank extraction controls were included in each DNA extraction procedure and subjected to amplification (Cooper & Poinar, 2000).

### 2.3 PCR amplification and sequencing

To assign the specimens to a clade and confirm their species identities, we amplified and sequenced a 182 bp fragment (141 bp without primers) of the mitochondrial cytochrome *b* gene that spans positions 13,881 to 14,062 in the short‐tailed albatross mitochondrial genome (all positions in this study are numbered according to the short‐tailed albatross reference mitochondrial genome [GenBank accession number: NC_026190] (Lounsberry et al., 2015)). Previous studies have demonstrated that this region of cytochrome *b* can differentiate North Pacific albatross (*Phoebastria* spp.) species and short‐tailed albatross clades (Eda et al., 2006, 2012; Eda, Koike, & Higuchi, 2016). This fragment was amplified using a previously published forward primer, Lcyt.246.dio (5′‐CCTCCACGCAAACGGAG‐3′) (Eda et al., 2006), and a novel reverse primer, R427 (5′‐CTCAGAATGATATTTGTCCTCATG‐3′), designed with an alignment of North Pacific albatross cytochrome *b* sequences obtained from GenBank (Sayers et al., 2020). The efficiency and specificity of this primer pair were assessed using NetPrimer (http://www.premierbiosoft.com/netprimer) and Primer‐BLAST (Ye et al., 2012).

All PCR amplifications and post‐PCR procedures were conducted in a negatively pressured laboratory that is physically separated from the aDNA laboratory. PCRs were performed on a Mastercycler Personal or Gradient thermal cycler (Eppendorf, Mississauga, Ontario, Canada) in a 30–40 μl reaction volume consisting of 1.5× PCR Gold Buffer (Applied Biosystems, Austin, Texas, United States of America), 2 mM MgCl_2_, 0.2 mM of each dNTP, 0.3 μM of each primer, 1 mg/mL BSA, 1–4.5 μl DNA sample, and 0.75–1.5 U AmpliTaq Gold (Applied Biosystems, Austin, Texas, United States of America). The thermal cycling program for the PCRs consisted of an initial denaturation step at 95°C for 12 min followed by 60 cycles at 95°C for 30 s (denaturation), 52–54°C for 30 s (annealing), and 70°C for 40 s (extension), and a final extension step at 72°C for 7 min. In order to detect instances of contamination, a negative PCR control was included in each PCR run (Cooper & Poinar, 2000).

To determine whether DNA was successfully amplified, 5 μl of PCR product from each specimen was pre‐stained with SYBR Green I (Invitrogen, Waltham, Massachusetts, United States of America), electrophoresed on a 2% agarose gel, and visualized with a Dark Reader transilluminator (Clare Chemical Research, Dolores, Colorado, United States of America). Successfully amplified specimens were directly sequenced with the amplification primers in the forward and/or reverse direction at Eurofins Genomics. Prior to sequencing, some of the specimens were purified with ExoSAP‐IT Express (Affymetrix, Santa Clara, California, United States of America) following the manufacturer's protocols. As an additional contamination control measure and to ensure that any observed polymorphisms were not the result of post‐mortem damage, DNA extraction and/or amplification were repeated for all of the specimens (Cooper & Poinar, 2000; Winters et al., 2011). In total, DNA extraction was repeated once for 20 of the specimens (two DNA extractions in total) and twice for three of the specimens (three DNA extractions in total) (Table S1).

### 2.4 Sequence analysis

The sequences obtained from each specimen were visually examined, trimmed in order to remove the primer sequences, and assembled into a consensus sequence with ChromasPro version 2.1.8 (http://technelysium.com.au/wp/chromaspro). The consensus sequences obtained from each specimen are 141 bp long and span positions 13,898 to 14,038 in the short‐tailed albatross reference mitochondrial genome. All of the cytochrome *b* consensus sequences have been deposited in GenBank under accession numbers OK077943–OK077985.

Oftentimes, the bones of North Pacific albatross species are difficult to distinguish using conventional morphology‐ or metric‐based zooarchaeological approaches (Eda et al., 2006; Porcasi, 1999b). Consequently, we confirmed the morphology‐based identifications of the specimens as short‐tailed albatrosses by comparing the obtained cytochrome *b* sequences to reference sequences whose taxonomic identity is known. We first compared each specimen's cytochrome *b* sequence to reference sequences accessioned in GenBank (Sayers et al., 2020) through BLASTn (Altschul et al., 1990) searches in order to determine their closest taxonomic match. Subsequently, multiple alignments of the sequences from the Yuquot albatross specimens and published reference specimens from Laysan (*Phoebastria immutabilis*), Black‐footed (*Phoebastria nigripes*), and Waved (*Phoebastria irrorata*), as well as Clade 1 and Clade 2 short‐tailed albatrosses (Eda et al., 2008, 2010; Lounsberry et al., 2015; Nunn et al., 1996; Walsh & Edwards, 2005) were performed through ClustalW (Thompson et al., 1994) as implemented in BioEdit version 7.2.5 (Hall, 1999). The resulting alignment was examined in BioEdit and the sequences were trimmed to the same length. Variable positions within the analyzed fragment were then identified and extracted using the “Show variable sites only” tool in FaBox version 1.61 (Villesen, 2007). A species identification was assigned to a specimen if both the BLASTn search and the examination of the aligned sequences indicated its sequence matched or closely resembled homologous sequences from a single species and differed significantly from closely related species. To assign the short‐tailed albatross specimens to a clade, we examined the sequence alignment we constructed and determined which clade‐specific mutations each specimen exhibited. Within the analyzed cytochrome *b* fragment, Clades 1 and 2 short‐tailed albatrosses can be differentiated by a single nucleotide polymorphism (SNP) at position 13,923. At this position, Clade 1 individuals exhibit a T, while Clade 2 individuals exhibit a C (Eda et al., 2006).

### 2.5 Statistical analysis

All statistical analyses were conducted using PAST version 4.05 (Hammer et al., 2001). Two‐tailed Fisher's exact tests (Mehta & Patel, 1983) were used to compare amplification success rates and haplotype frequencies between stratigraphic zones at Yuquot. Comparisons of the haplotype frequencies observed among short‐tailed albatross from Yuquot, the Funadomari and Hamanaka 2 archaeological sites on Rebun Island, Japan, and the Bentenjima archaeological site on Bentenjima, Japan (Eda et al., 2012; Eda, Koike, & Higuchi, 2016) (Figure 1) (Table 1), were performed with a two‐tailed Fisher's exact test (Mehta & Patel, 1983). When significant differences in haplotype frequencies or amplification success rates were detected between stratigraphic zones or archaeological sites, post‐hoc pairwise two‐tailed Fisher's exact tests (Mehta & Patel, 1983) with a Bonferroni correction (MacDonald & Gardner, 2000; McDonald, 2014) were used to identify pairs of groups that significantly differed. A significance level of *α* = .05 was used for all statistical analyses.

**TABLE 1:** Haplotype frequencies among short‐tailed albatrosses from archaeological sites in the Northwest Pacific.

Site	Clade 1	Clade 2	Haplotype 3	Reference
Bentenjima	7	7	0	Eda, Koike, and Higuchi (2016)
Funadomari	8	7	0	Eda, Koike, and Higuchi (2016)
Hamanaka 2	14	28	0	Eda et al., 2012

## 3 RESULTS

### 3.1 PCR amplification and sequencing

The targeted 182 bp fragment of cytochrome *b* was successfully amplified from 43 of the 51 (84.3% success rate) specimens. Amplification success by stratigraphic zone ranged from 66.7% (Zone 1) to 94.4% (Zone III) (Table 2), with a mean success rate of 82.5%. No significant difference in amplification success was observed between stratigraphic zones (two‐tailed Fisher's exact test, *p =* .27554). No DNA was amplified from any of the blank extraction or negative PCR controls.

**TABLE 2:** Amplification success by stratigraphic zone

Stratigraphic Zone	Amplification successful	Amplification failure	Total
1	2 (66.7%)	1 (33.3%)	3
2	16 (76.2%)	5 (23.8%)	21
3	17 (94.4%)	1 (5.6%)	18
4	8 (88.9%)	1 (11.1)	9
Total	43 (84.3%)	8 (15.7%)	51

The sequences obtained from all 43 of the specimens that yielded DNA were successfully reproduced through repeat amplification and/or DNA extraction. However, in some instances, sequences obtained from the same sample differed by C → T and G → A transitions. As they could not be replicated through repeat extraction and/or repeat amplification and sequencing (Winters et al., 2011), these transitions likely reflect post‐mortem cytosine deamination (See Dabney et al., 2013 for review.). Messy or primate sequences were occasionally obtained from some of the specimens, which likely reflects the amplification of contaminant DNA. However, through repeat amplification and sequencing, we were able to obtain clear albatross sequences from these specimens.

### 3.2 Species identification

The results of the BLASTn searches indicated that all of the specimens have cytochrome *b* sequences that most closely resemble short‐tailed albatross reference sequences (≥99.29% sequence similarity, 100% query coverage). The maximum sequence difference observed between our specimens and short‐tailed albatross reference sequences (1.42%, 100% query coverage) did not exceed the minimum sequence difference between our specimens and other North Pacific albatross species (2.13%, 100% query coverage). An examination of the sequence alignment we constructed revealed that all of the specimens have SNPs consistent with them being short‐tailed albatross (Table *3*). For instance, all of the specimens exhibited a T at position 13,920, which is an SNP that is unique to short‐tailed albatross (Table *3*). Together these data support the morphology‐based identification of these 43 specimens as short‐tailed albatross.

**TABLE 3:** Variable nucleotide positions in the analyzed 141 bp fragment of the mitochondrial cytochrome *b* gene

Species/Specimen	GenBank Accession Numbe	Variable Nucleotide Positions														
		13,911	13,920	13,923	13,926	13,935	13,941	13,962	13,971	13,972	13,980	13,990	14,001	14,007	14,034	14,037
*P*. *albatrus* Clade 1	NC_026190	C	T	T	T	C	A	G	A	A	C	C	C	C	T	G
ALB1		.	.	.	.	.	.	.	.	.	.	.	.	.	.	.
ALB3		.	.	.	.	.	.	.	.	.	.	.	.	.	.	.
ALB7		.	.	.	.	.	.	.	.	.	.	.	.	.	.	.
ALB8		.	.	.	.	.	.	.	.	.	.	.	.	.	.	.
ALB9		.	.	.	.	.	.	.	.	.	.	.	.	.	.	.
ALB10		.	.	.	.	.	.	.	.	.	.	.	.	.	.	.
ALB11		.	.	.	.	.	.	.	.	.	.	.	.	.	.	.
ALB13		.	.	.	.	.	.	.	.	.	.	.	.	.	.	.
ALB14		.	.	.	.	.	.	.	.	.	.	.	.	.	.	.
ALB15		.	.	.	.	.	.	.	.	.	.	.	.	.	.	.
ALB16		.	.	.	.	.	.	.	.	.	.	.	.	.	.	.
ALB18		.	.	.	.	.	.	.	.	.	.	.	.	.	.	.
ALB19		.	.	.	.	.	.	.	.	.	.	.	.	.	.	.
ALB20		.	.	.	.	.	.	.	.	.	.	.	.	.	.	.
ALB21		.	.	.	.	.	.	.	.	.	.	.	.	.	.	.
ALB22		.	.	.	.	.	.	.	.	.	.	.	.	.	.	.
ALB23		.	.	.	.	.	.	.	.	.	.	.	.	.	.	.
ALB24		.	.	.	.	.	.	.	.	.	.	.	.	.	.	.
ALB25		.	.	.	.	.	.	.	.	.	.	.	.	.	.	.
ALB26		.	.	.	.	.	.	.	.	.	.	.	.	.	.	.
ALB27		.	.	.	.	.	.	.	.	.	.	.	.	.	.	.
ALB28		.	.	.	.	.	.	.	.	.	.	.	.	.	.	.
ALB29		.	.	.	.	.	.	.	.	.	.	.	.	.	.	.
ALB30		.	.	.	.	.	.	.	.	.	.	.	.	.	.	.
ALB33		.	.	.	.	.	.	.	.	.	.	.	.	.	.	.
ALB34		.	.	.	.	.	.	.	.	.	.	.	.	.	.	.
ALB35		.	.	.	.	.	.	.	.	.	.	.	.	.	.	.
ALB36		.	.	.	.	.	.	.	.	.	.	.	.	.	.	.
ALB39		.	.	.	.	.	.	.	.	.	.	.	.	.	.	.
ALB40		.	.	.	.	.	.	.	.	.	.	.	.	.	.	.
ALB41		.	.	.	.	.	.	.	.	.	.	.	.	.	.	.
ALB42		.	.	.	.	.	.	.	.	.	.	.	.	.	.	.
ALB44		.	.	.	.	.	.	.	.	.	.	.	.	.	.	.
ALB45		.	.	.	.	.	.	.	.	.	.	.	.	.	.	.
ALB46		.	.	.	.	.	.	.	.	.	.	.	.	.	.	.
ALB47		.	.	.	.	.	.	.	.	.	.	.	.	.	.	.
ALB48		.	.	.	.	.	.	.	.	.	.	.	.	.	.	.
ALB49		.	.	.	.	.	.	.	.	.	.	.	.	.	.	.
ALB50		.	.	.	.	.	.	.	.	.	.	.	.	.	.	.
ALB51		.	.	.	.	.	.	.	.	.	.	.	.	.	.	.
*P*. *albatrus* Clade 2	AB276047	.	.	C	.	.	.	.	.	.	.	.	.	.	.	.
ALB5		.	.	C	.	.	.	.	.	.	.	.	.	.	.	.
ALB43		.	.	C	.	.	.	.	.	.	.	.	.	.	.	.
ALB32		T	.	C	.	.	.	.	.	.	.	.	.	.	.	.
*P*. *immutabilis*	NC_026189	.	C	.	C	.	.	.	.	.	.	.	.	.	C	.
*P*. *immutabilis*	AB276048	.	C	C	C	.	.	.	.	.	.	.	.	.	C	.
*P*. *immutabilis*	AB276049	.	C	.	C	.	.	.	.	.	.	.	T	.	C	.
*P*. *irrorata*	U48951	.	C	.	.	.	G	.	G	G	T	A	.	.	C	.
*P*. *nigripes*	NC_026188	.	C	.	.	.	.	.	G	.	.	.	.	T	C	.
*P*. *nigripes*	AB276051	.	C	.	.	.	.	A	G	.	.	.	.	T	C	A
*P*. *nigripes*	AB426115	.	C	.	.	.	.	A	G	.	.	.	.	T	.	A
*P*. *nigripes*	AY641402	.	C	.	.	T	.	A	G	.	.	.	.	T	C	A

*Notes*: Positions are numbered according to the short‐tailed albatross reference mitochondrial genome (GenBank accession number: NC_026190) (Lounsberry et al., 2015). Dots indicate positions identical to the short‐tailed albatross reference mitochondrial genome. ALB# denotes archaeological specimens analyzed in this study.

### 3.3 Clade identification

Comparisons with published short‐tailed albatross cytochrome *b* haplotypes associated with Clade 1 and Clade 2 indicated 40 (93%) of the specimens belong to Clade 1 (Table 3). The Clade 1 haplotype was the most common haplotype in each of the stratigraphic zones represented at Yuquot (Table *4*). Two of the remaining three specimens exhibit the Clade 2 haplotype (Table 3). The remaining specimen (ALB32) has a cytochrome *b* haplotype that has not been previously documented among modern or archaeological short‐tailed albatrosses. In addition to exhibiting the C at position 13,923 that characterizes the Clade 2 haplotype, this specimen exhibited an additional synonymous C → T transition at position 13,911 (Table 3). Here, we refer to this novel haplotype as Haplotype 3. Albatrosses exhibiting the Clade 2 haplotype and Haplotype 3 were only identified in Zone II (Table 4). No significant differences in haplotype frequencies were observed between any of the stratigraphic zones at Yuquot (two‐tailed Fisher's exact test, *p =* .31632).

**TABLE 4:** Short‐tailed albatross haplotype frequencies by stratigraphic zone

Stratigraphic Zone	Clade 1	Clade 2	Haplotype 3	Total
1	2 (100%)	0 (0%)	0 (0%)	2
2	13 (81.3%)	2 (12.5%)	1 (6.2%)	16
3	17 (100%)	0 (0%)	0 (0%)	17
4	8 (100%)	0 (0%)	0 (0%)	8
Total	40 (93%)	2 (4.7%)	1 (2.3%)	43

The cytochrome *b* haplotype frequencies observed at Bentenjima, Funadomari, Hamanaka 2, and Yuquot, significantly differ (two‐tailed Fisher's exact test, *p* < .001). A post‐hoc analysis indicates the haplotype frequencies observed at Yuquot significantly differ from those observed at each of the Japanese archaeological sites (pairwise two‐tailed Fisher's exact tests with a Bonferroni correction, all *p* < .001). No significant difference in haplotype frequencies was observed between any of the Japanese archaeological sites (pairwise two‐tailed Fisher's exact tests with a Bonferroni correction, all *p* > .05).

## 4 DISCUSSION

### 4.1 Authenticity of ancient DNA data and DNA preservation

The quantity and quality of endogenous DNA preserved within ancient and historical remains is typically low, making them susceptible to contamination with modern DNA and amplification products (Cooper & Poinar, 2000; Yang & Watt, 2005). However, when taken together, various lines of evidence suggest we obtained authentic aDNA sequences from the archaeological short‐tailed albatross specimens from Yuquot that we examined. First, no DNA was amplified from any of the blank extraction or negative PCR controls, indicating systematic contamination did not occur (Cooper & Poinar, 2000). Second, the sequences obtained from each specimen were successfully reproduced through repeat DNA extractions and/or PCR amplifications (Cooper & Poinar, 2000). Third, some of the sequences obtained from a handful of the the specimens exhibited C → T and G → A transitions associated with cytosine deamination, a form of post‐mortem damage characteristic of aDNA (Dabney et al., 2013). Fourth, prior to DNA extraction, all of the remains were decontaminated through a combination of bleach and UV irradiation (Yang & Watt, 2005). Fifth, all pre‐PCR procedures were conducted in a dedicated aDNA laboratory physically separated from the post‐PCR laboratory (Cooper & Poinar, 2000).

Previous analyses of archaeological mammal (e.g., Moss et al., 2006) and fish remains (e.g., Cannon & Yang, 2006; Morin, Zhang, et al., 2021; Rodrigues et al., 2018; Speller et al., 2012; Yang et al., 2004) from coastal British Columbia have yielded similarly high‐amplification success rates for mitochondrial DNA, indicating DNA is often well‐preserved in faunal remains from the region. The exceptional DNA preservation observed at Yuquot and other sites along the coast of British Columbia likely partially reflects the region's mild year‐round temperatures being conducive to DNA preservation (Speller, 2018; Yang et al., 2004). The shell‐rich matrix at many sites in the region, including Yuquot (Dewhirst, 1980), may also contribute to DNA preservation by increasing soil alkalinity (Yang et al., 2004). Mild temperatures and neutral or alkaline conditions are both associated with reduced rates of hydrolytic depurination (Lindahl & Nyberg, 1972), which is hypothesized to be the chemical pathway primarily responsible for DNA fragmentation (Dabney et al., 2013).

### 4.2 Historical genetic structure of British Columbian short‐tailed albatrosses

Throughout its four millennia of occupation, the short‐tailed albatrosses foraging near Yuquot and harvested by its inhabitants predominately belonged to Clade 1. Individuals belonging to this clade represent between 81.3% and 100% of the specimens in each stratigraphic zone (Table 4). As genetic data from present‐day individuals are unavailable, it is unknown whether the short‐tailed albatross population in British Columbia continues to consist mainly of Clade 1 individuals (Eda, Izumi, et al., 2016; Kuro‐o et al., 2010). However, circumstantial evidence does suggest Clade 1 albatrosses continue to forage off British Columbia's coast. Satellite tracking data indicate British Columbian waters presently form part of the foraging range of birds from the Torishima breeding colony (Orben et al., 2018; Suryan et al., 2006, 2007). Today, the vast majority of short‐tailed albatrosses on Torishima belong to Clade 1 (Eda et al., 2011, 2012; Eda, Izumi, et al., 2016; Kuro‐o et al., 2010). Consequently, the presence of birds from Torishima in the province's waters does raise the possibility that Clade 1 albatrosses continue to forage along the coast of British Columbia. Nonetheless, confirming Clade 1’s continued use of British Columbian waters requires genetic data from modern individuals.

Although our data suggest the genetic structure of short‐tailed albatrosses at Yuquot was largely stable across time, our analysis did reveal some temporal changes in clade membership. Although absent in other stratigraphic zones, Clade 2 short‐tailed albatrosses and an individual with a novel haplotype (Haplotype 3) were identified in Zone II, which dates to between 1000 BC and 800 CE. The reason for this change is unknown. Further analyses of time series of albatross remains from other areas of British Columbia should be conducted in order to evaluate whether this change was local or regional in nature.

At present, Clade 1 and 2 short‐tailed albatrosses for the most part breed on different islands. As noted above, Clade 1 individuals presently only breed on Torishima, while Clade 2 individuals primarily breed on the Senkaku/Diaoyu/Diaoyutai Islands, and to a lesser extent Torishima (Eda et al., 2011, 2012; Eda, Izumi, et al., 2016; Kuro‐o et al., 2010). Given this inter‐clade segregation in breeding sites, it is tempting to speculate that the Clade 1 individuals represented at Yuquot are from Torishima, while the Clade 2 individuals originated from the Senkaku/Diaoyu/Diaoyutai Islands. However, we would caution against this interpretation. Since genetic data from other historical short‐tailed albatrosses breeding colonies are not available, it is not possible to ascertain whether these clades had similarly restrictive breeding distributions in the past. Identifying the possible natal site(s) of the Clade 1 and 2 short‐tailed albatrosses at Yuqout will therefore require genetic data from the other islands where the species formerly bred. In cases where archaeological, palaeontological, or taxidermied specimens from former breeding sites are unavailable, DNA preserved in sediments (sedaDNA) can potentially provide such data (e.g., Willerslev et al., 2003). Additional genetic data from current and former breeding colonies would also assist in clarifying the origin of the Haplotype 3 bird we identified. If Clade 1 and Clade 2 historically bred on multiple islands, determining the historical breeding colony affinities of British Columbian short‐tailed albatrosses would require sequencing a more variable region, such as the mitochondrial control region (e.g., Eda et al., 2012).

### 4.3 Spatial variation in the relative abundance of clade 1 and 2 short‐tailed albatrosses

The high relative frequency of Clade 1 birds and relative scarcity of Clade 2 birds at Yuquot contrasts with the patterns observed by previous analyses of archaeological short‐tailed albatrosses. Ancient DNA analyses of short‐tailed albatross remains from archaeological sites located in the Northwest Pacific have found both Clades 1 and 2 to be well represented (Eda et al., 2006, 2012; Eda, Koike, et al., 2016). At the Late Jomon (c. 3800–3500 BP) Funadomari and Okhotsk culture (c. 1000–1200 BP) Bentenjima sites, the relative frequency of Clade 1 and Clade 2 birds is equal or nearly equal (Table 1) (Eda, Koike, & Higuchi, 2016). At the Okhotsk culture (c. 1200–850 BP) Hamanaka 2 site, Clade 2 birds predominate, but Clade 1 birds are well represented (Table 1) (Eda et al., 2012).

The significant differences in haplotype frequencies we observed between Yuquot and these Japanese sites could potentially reflect their inhabitants using different hunting strategies. However, we are not aware of any ecological, behavioral, or morphological differences that would make these two clades susceptible to different hunting strategies. As such, we hypothesize this spatial variation in haplotype frequencies reflects historical differences in the distribution of Clade 1 and 2 short‐tailed albatrosses. While the results of previous studies indicate both clades were historically abundant in the Northwest Pacific (Eda et al., 2006, 2012; Eda, Koike, & Higuchi, 2016), our data indicate Clade 1 historically foraged in the waters around Vancouver Island to a greater extent. Determining whether Clade 1 historically made more extensive use of other areas along the Pacific Coast of North America requires data from historical and/or archaeological specimens from additional locales. However, genetic analyses of taxidermied specimens from Cook Inlet (*n* = 1) and St. Paul Island (*n* = 1), Alaska, and a single archaeological specimen from San Miguel Island, California, similarly only identified Clade 1 birds (Nisan, 2014). These data suggest Clade 1 birds did indeed forage in other areas of the Northeast Pacific. If supported by additional data, Clade 1 and 2 short‐tailed albatrosses having differing distributions would lend support to Eda and colleagues' (Eda et al., 2020; Yamasaki et al., 2022) argument that these two clades represent distinct species.

Our findings are consistent with the results of previous stable carbon (*δ* ^13^C) and nitrogen (*δ* ^15^N) isotope analyses of bone collagen from archaeological short‐tailed albatrosses. At the Hamanaka 2 site, Eda et al. (2012) found a very small (~0.5‰) but statistically significant difference between the *δ* ^15^N values of bone collagen from Clade 1 and Clade 2 individuals. The main driver of isotopic variation among short‐tailed albatrosses is thought to be the degree to which individuals foraged in areas with different isotopic baselines, rather than inter‐individual differences in diet content (Vokhshoori et al., 2019). As such, this significant difference in *δ* ^15^N values between Clade 1 and 2 individuals at Hamanaka 2—like our data—suggests these two clades may have had differing foraging ranges. Comparisons of archaeological short‐tailed albatrosses from different regions have similarly revealed population‐level variation in migratory behavior. Interpreting compound‐specific as well as ‘bulk’ bone collagen *δ* ^13^C and *δ* ^15^N data, Vokhshoori et al. (2019) found albatrosses from California foraged to a greater extent in the California Current System than Japanese or Russian birds. More recently, analyses of *δ* ^13^C and *δ* ^15^N values from short‐tailed albatrosses from Yuquot indicate they exhibit significantly less isotopic variation than archaeological populations from Japan, Russia, Oregon, and California (Guiry et al., 2022). Guiry et al. (2022) interpret this low degree of isotopic variation to indicate that the albatrosses recovered from Yuquot likely had a high degree of foraging site fidelity relative to the individuals analyzed from other regions, with individuals returning year after year and generation after generation to forage near the site.

#### 4.3.1 Sustainability of the short‐tailed albatross harvest at Yuquot

At Yuquot, short‐tailed albatrosses are the most abundant avian species in each of the site's four stratigraphic zones, indicating that they were the focus of bird hunting activities throughout its occupation (McAllister, 1980). Although hunted intensively relative to other birds, there is little evidence that the short‐tailed albatross population exploited by the Yuquot Mowachaht experienced resource depression. Across the site's four stratigraphic zones, the abundance of short‐tailed albatross relative to other avifauna is fairly consistent (Range: 26.7%–39.6%, *x̄* = 32.2%, *s* = .06) (McAllister, 1980), with no obvious temporal trends in the taxon's relative abundance. Stable isotope analyses similarly indicate the migratory behavior of short‐tailed albatrosses from Yuquot remained relatively stable, with individuals across time exhibiting a comparable high degree of site fidelity (Guiry et al., 2022). Guiry et al. (2022) suggest this stability in migratory behavior indicates the albatross population foraging in the vicinity of Yuquot was not overexploited.

The genetic data we have collected in this study provide additional corroboration for the sustainability of the short‐tailed albatross harvest carried out by the Yuquot Mowachaht. Among other taxa, aDNA analyses have demonstrated that by altering competition levels, overhunting and other anthropogenic disturbances can result in biological turnover events that see the total or partial replacement of one genetic lineage with another (e.g., Boessenkool et al., 2009; Collins et al., 2014; Grosser et al., 2016; Rawlence et al., 2015, 2017). At Yuquot, there is little evidence that the harvesting of short‐tailed albatrosses led to such a biological turnover event. Instead, the lack of significant inter‐zone differences in haplotype frequencies and dominance of Clade 1 throughout the site's occupation suggest a high degree of population continuity over the last four millennia. The sustainability of the Yuquot Mowachaht short‐tailed albatross harvest as evinced by zooarchaeological, isotopic, and genetic data contributes to the growing body of evidence for the sustainable exploitation of marine resources by Indigenous peoples in Northwestern North America over centuries to millennia (e.g., McKechnie et al., 2014; Morin, Royle, et al., 2021; Morin, Zhang, et al., 2021).

## 5 SUMMARY AND CONCLUSION

This study represents the most extensive genetic analysis of short‐tailed albatross in the Northeast Pacific conducted to date and has provided insights into this species' historical biogeography. Our results indicate that for the past four millennia most of the short‐tailed albatrosses foraging off the west coast of Vancouver Island belonged to Clade 1. Between 1000 BCE and 800 CE, Clade 2 albatrosses and an albatross with previously undocumented haplotype also foraged in the vicinity of Yuquot to a limited degree. This sharply contrasts with the pattern observed at three archaeological sites in northern Japan, where Clade 1 and Clade 2 are more equally represented. This spatial variation in the relative abundance of these two clades may suggest the distribution of Clades 1 and 2 historically differed. While both albatross clades foraged extensively in the Northwest Pacific, Clade 1 albatrosses appear to have foraged along the west coast of Vancouver Island to a greater extent. It is expected that genetic analyses of albatrosses from other archaeological sites along the west coast of North America will continue to enhance our understanding of the historical distribution of these clades.

Our finding that the distribution of Clade 1 and 2 short‐tailed albatrosses may have historically differed has significant implications for the conservation of this species. If the inter‐clade distributional difference we observed persists or re‐emerges with population recovery, the degree to which these two clades are exposed to certain threats may vary (cf. Suryan et al., 2007). Due to its historical predominance along the North American coast, documented or potential threats occurring within North American waters, in particular, may pose a greater risk to Clade 1 albatrosses. Within North American waters, recent assessments have highlighted continued or potential mortality from incidental catches by hook‐and‐line groundfish fisheries and the heightened potential for oil spills associated with projected increases in shipping traffic as threats to the species (COSEWIC, 2013; Fox et al., 2021; U.S. Fish and Wildlife Service, 2020). Ensuring the recovery of both Clades 1 and 2 will therefore require conservation plans that account for the unique threats these clades face as a result of their potentially distinct distributions.

## AUTHOR CONTRIBUTIONS

**Thomas C. A. Royle:** Conceptualization (equal); data curation (lead); formal analysis (lead); investigation (lead); methodology (lead); project administration (supporting); supervision (supporting); validation (lead); visualization (lead); writing – original draft (lead); writing – review and editing (lead). **Eric J. Guiry:** Conceptualization (equal); resources (equal); writing – review and editing (supporting). **Hua Zhang:** Investigation (supporting); supervision (supporting); validation (supporting); writing – review and editing (supporting). **Lauren T. Clark:** Investigation (supporting); validation (supporting); writing – review and editing (supporting). **Shalegh M. Missal:** Investigation (supporting); validation (supporting). **Sophie A. Rabinow:** Investigation (supporting); validation (supporting); writing – review and editing (supporting). **Margaretta James:** Resources (supporting); writing – review and editing (supporting). **Dongya Y. Yang:** Conceptualization (equal); funding acquisition (lead); methodology (supporting); project administration (lead); resources (equal); supervision (lead); writing – review and editing (supporting).

## CONFLICT OF INTEREST

The authors declare no competing interests.

## Supporting information


Table S1

## Data Availability

All of the DNA consensus sequences have been deposited in GenBank (accession numbers OK077943–OK077985).
